# Granulocyte colony-stimulating factor priming improves embryos and pregnancy rate in patients with poor ovarian reserve: a randomized controlled trial

**DOI:** 10.1186/s12958-023-01082-w

**Published:** 2023-03-21

**Authors:** Masao Jinno, Yukoku Tamaoka, Koji Teruya, Aiko Watanabe, Naohisa Hatakeyama, Tomoya Goda, Hayato Kimata, Yuichi Jinno

**Affiliations:** 1 Women’s Clinic Jinno, 3-11-7 Kokuryou-Chou, Choufu City, Tokyo, 182-0022 Japan; 2 Ikebukuro Metropolitan Clinic, Toshima-Ku, Tokyo, 171-0021 Japan; 3 Department of Obstetrics and Gynecology, Inagi Municipal Hospital, Inagi City, Tokyo, 206-0801 Japan; 4 Faculty of Health Sciences, Kyorin University, Mitaka City, Tokyo, 181-8612 Japan

**Keywords:** Granulocyte colony-stimulating factor (G-CSF), Assisted reproductive technology, Pregnancy rate, Oocyte developmental competence, Diminished ovarian reserve, Anti-Müllerian hormone, Preantral follicle growth

## Abstract

**Background:**

Granulocyte colony-stimulating factor (G-CSF) administration increased ovarian preantral follicles and anti-Müllerian hormone (AMH) in animal models with diminished ovarian reserve. We investigated whether G-CSF priming before treatment with assisted reproductive technology (ART) improved embryo development and pregnancy rate while increasing serum AMH in patients with poor ovarian reserve.

**Methods:**

In this prospective randomized open-label controlled trial, 100 patients 20 to 42 years old with AMH below 2 ng/mL were randomized to priming or control groups (50 patients each). None had over 1 ART failure, day-3 follicle-stimulating hormone (FSH) above 30 IU/L, uterine anomalies, or a partner with azoospermia. All patients initially underwent conventional infertility treatment for 2 consecutive cycles in which the priming group but not controls received a subcutaneous G-CSF priming injection during the early luteal phase. Each group then underwent 1 cycle of in vitro fertilization/intracytoplasmic sperm injection and fresh embryo transfer (IVF/ICSI-fresh ET), followed by cryopreserved ET if needed until live birth or embryo depletion. AMH was measured before and after priming.

**Results:**

Fertilization rate, embryonic development, and implantation rate by fresh ET were significantly improved by priming. Clinical and ongoing pregnancy rates by IVF/ICSI-fresh ET were significantly higher with priming (30% and 26% in 47 ART patients; 3 delivered with conventional treatment) than in controls (12% and 10% in 49 ART patients; 1 dropped out). With priming, significantly more patients achieved cryopreservation of redundant blastocysts. The cumulative live birth rate was 32% in 50 patients with priming, significantly higher than 14% in 49 controls (relative risk, 2.8; 95% confidence interval, 1.04–7.7). Infants derived from priming had no congenital anomalies, while infant weights, birth weeks, and Apgar scores were similar between groups. Among 4 variables (age, day-3 FSH, AMH, and priming), logistic regression significantly associated age and priming with cumulative live birth. Priming significantly increased serum AMH. No adverse effects of priming were observed.

**Conclusion:**

G-CSF priming improved embryonic development and pregnancy rate during ART treatment and increased AMH in patients with poor ovarian reserve. Enhanced preantral follicle growth likely was responsible.

**Trial registration:**

UMIN registration in Japan (UMIN000013956) on May 14, 2014. https://www.umin.ac.jp/ctr/index.htm.

## Background

Ten years ago, we treated repeated implantation failure in 10 women with diminished ovarian reserve by administering granulocyte colony-stimulating factor (G-CSF) in association with embryo transfers (ET), based upon previous reports [1, 2]. No short-term outcome improvements resulted, but serendipitously 3 of the 10 spontaneously became pregnant 2 months later. One of these pregnancies involved twins in a 45-year-old woman without ovulation induction. We hypothesized that G-CSF administration might have stimulated preantral follicle growth, with improved ovulation after 2 months.

In diabetic rats, administration of G-CSF consistently decreased ovarian follicular degeneration as well as degeneration and fibrosis of ovarian stroma, while increasing serum anti-Müllerian hormone (AMH) concentrations [3]. G-CSF administration also significantly increased ovarian preantral follicles and serum AMH in rats with diminished ovarian reserve induced by cisplatin [4].

Considering our experience with the 3 patients and these G-CSF effects in animal models, we designed the present prospective randomized clinical trial examining whether G-CSF administration in the early luteal phases of each of 2 cycles (G-CSF priming) preceding the cycle involving assisted reproductive technology (ART) improved embryonic development and pregnancy rate following ART in patients with poor ovarian reserve, as well as whether priming increased serum AMH concentrations. We also investigated associations between effects of G-CSF priming and killer-cell immunoglobulin-like receptor (KIR) types, since some types have been associated with improved implantation following G-CSF administration on the day of ET [1].

## Materials and methods

### Study design

**Fig. 1:**
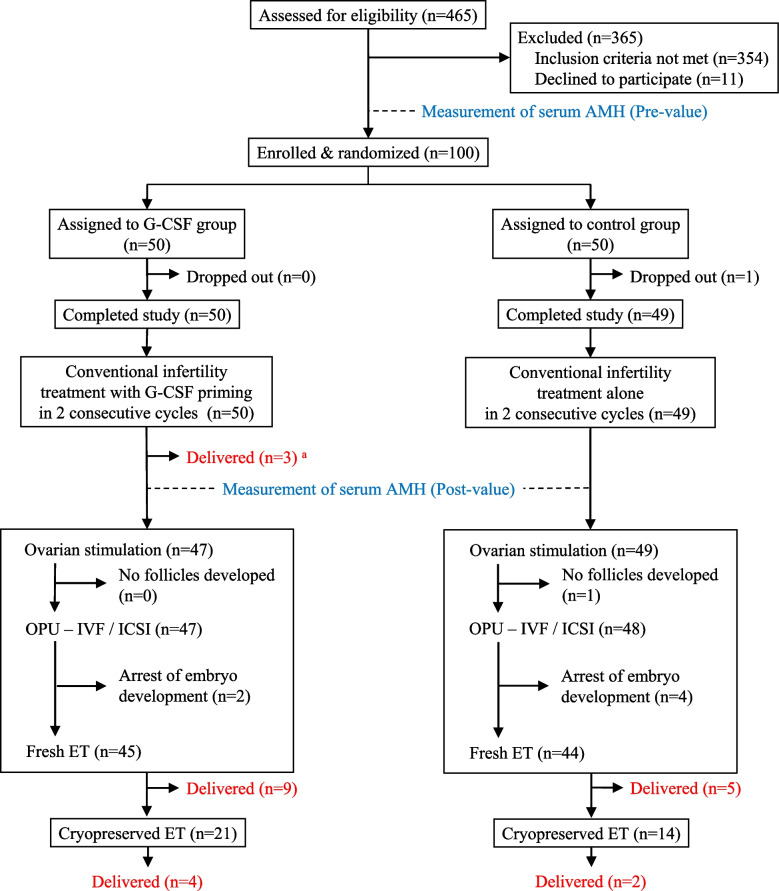
Participant flow diagram. The rate of live delivery among patients was 32% (16/50) in the G-CSF group, significantly higher than 14% (7/49) in controls (chi-squared test). G-CSF, granulocyte colony-stimulating factor; OPU, oocyte pick-up; IVF, in vitro fertilization; ICSI, intracytoplasmic sperm injection; ET, embryo transfer. ^a^ All 3 of these patients conceived with spontaneous ovulation following menstruation after the initial administration of G-CSF

Our prospective open-label randomized clinical trial investigated whether G-CSF priming preceding ART enhanced preantral follicle growth, thus increasing the ART pregnancy rate in patients with poor ovarian reserve. Between May 19, 2014 and November 26, 2018, a total of 465 patients sought ART treatment at Women’s Clinic Jinno; 111 met study inclusion criteria. Eleven declined participation, leaving 100 to be enrolled and randomly assigned to groups undergoing or not undergoing G-CSF priming prior to standard ART. Randomization involved patients drawing from a box containing group assignments in sealed envelopes mixed 1:1 (Fig. 1). Neither patients nor investigators were blinded to resulting assignments.

To more clearly detect G-CSF-related improvement of ovarian reserve, we limited our study to patients with mildly to moderately decreased ovarian reserve, excluding patients with severe diminution. We chose a serum AMH concentration lower than 2 ng/mL as an inclusion criterion for our study, given that 93% of infertile women in their forties have been found to have values below 2 ng/mL [5]. An earlier study found 1.9 ng/mL to be the median serum AMH concentration among nulliparous 38-year-old Japanese women, a population on the verge of a precipitous decline in fertility [6].

Based on such considerations, our inclusion criteria were age between 20 and 42 years; no more than 1 prior oocyte retrieval attempt; serum AMH concentration below 2 ng/mL; day-3 serum FSH concentration below 30 IU/L; a medical history free of serious allergic disease, severe hepatic, renal, or heart disease, or uterine infertility; and a male partner without azoospermia.

All enrolled patients initially received conventional infertility treatments in 2 consecutive cycles. (Since no patient in either group had bilateral tubal occlusion, we believed that a chance of pregnancy using conventional treatment existed.) During these 2 cycles the G-CSF group, but not the control group, underwent subcutaneous administration of G-CSF (100 μg of lenograstim; Neutrogin, Chyugai Pharmaceuticals, Tokyo, Japan) during the early luteal phase, based upon basal body temperature records, vaginal ultrasonographic findings, and, if necessary, serum progesterone determinations (Fig. 1). Controls received no placebo. Conventional infertility treatments included sexual intercourse or intrauterine insemination with or without ovarian stimulation by clomiphene citrate or a recombinant FSH regimen.

Both groups consisted of patients who had failed to conceive with conventional infertility treatments and then underwent ART using controlled ovarian stimulation. Embryos were transferred 2, 3, or 5 days after in vitro fertilization (IVF) or intracytoplasmic sperm injection (ICSI). Remaining embryos were cryopreserved at the blastocyst stage. When IVF/ICSI and fresh embryo transfer (ET) failed to result in delivery, cryopreserved embryos were thawed and transferred (cryopreserved ET) in subsequent spontaneous cycles. During actual ART treatment using fresh or cryopreserved ET, neither group received G-CSF.

Clinical pregnancy and ongoing pregnancy were diagnosed by ultrasonographic detection of a gestational sac and fetal heart movements, respectively. Abortion and delivery were defined as pregnancy loss before 22 weeks and birth after 22 weeks, respectively. Clinical pregnancy rates by IVF/ICSI-fresh ET (per ovarian stimulation) were compared between groups as the primary outcome measure. Cumulative live delivery rates, follicular growth, fertilization, and embryonic development were compared between groups as secondary outcome measures.

To elucidate stimulatory effects of G-CSF priming on preantral follicle growth, pre- *vs.* post-treatment changes in serum AMH concentration were compared between groups. Serum samples were obtained within 4 months preceding study enrollment and just before initiating ovarian stimulation for ART (Fig. 1).

To achieve a power of 0.8 and an α error of 0.05, the minimum number of participants required to identify a difference between hypothetical pregnancy rates of 10% and 25% in control and G-CSF groups was 113 patients per group, so we planned to recruit those numbers. However, interim assessment halfway through the expected trial duration already showed statistically significant benefit from G-CSF priming that was greater than expected. We therefore ended our trial with 50 enrollees per group, considering the ethical importance of offering potential benefits of G-CSF priming to control patients in a timely manner.

Informed consent was obtained from all patients. The study was approved by the Ethics Committees of both the Inagi Municipal Hospital and Women’s Clinic Jinno, and registered with the UMIN in Japan (UMIN000013956).

### Assisted reproductive technology

Follicular development was stimulated with the long protocol as described previously [7]. Briefly, buserelin acetate (Buserecur; Fuji Pharmaceuticals, Tokyo, Japan) at 900 µg per day was administered nasally from the mid-luteal phase until hCG administration. Human menopausal gonadotropin, (hMG, 300 IU i.m.; Ferring Pharmaceuticals, Tokyo, Japan) was administered daily from day 3. Human chorionic gonadotropin (hCG, 10 000 IU i.m.; Mochida Pharmaceuticals, Tokyo, Japan) was administered when the dominant follicle reached a diameter of 19 mm.

Oocytes were collected transvaginally 36 h after hCG administration and inseminated as described previously [8]. ICSI was performed when the male partner had severe infertility (sperm count < 5 × 10^6^ per mL and/or motility < 20%). Oocytes were considered fertilized when 2 pronuclei were observed 17 to 19 h after insemination or ICSI. At 2, 3, or 5 days after oocyte retrieval, embryos were transferred to the uterus according to number and quality of developing embryos for each patient. Progesterone (25 mg i.m.) was administered daily after ET.

Redundant embryos were cultured for 5 to 6 days after IVF/ICSI to the blastocyst stage and cryopreserved using a vitrification method. Thawed cryopreserved blastocysts were transferred to uteri on luteal day 5 of a spontaneous natural cycle, as described previously [7]. For luteal support, 5000 IU of hCG was administered on luteal days 5, 7, and 9.

### Evaluations of hormones and killer-cell immunoglobulin-like receptor genotypes

Serum concentrations of AMH, follicle-stimulating hormone (FSH), luteinizing hormone (LH), 17β-estradiol (E_2_), prolactin (PRL), testosterone (T), thyroid-stimulating hormone (TSH), free thyronine (FT_3_), free thyroxine (FT_4_), and dehydroepiandrosterone sulfate (DHEA-S) were measured by enzyme chemiluminescent immunoassays on cycle day 3 within 3 months before study enrollment.

Sensitivities and intra- and interassay coefficients of variation were 0.01 ng/mL (1.4%, 0.8%) for AMH, 0.06 IU/L (2.3%, 1.0%) for FSH, 0.11 IU/L (6.6%, 3.4%) for LH, 5.0 pg/mL (0.7%, 0.9%) for E_2_, 0.10 ng/mL (1.2%, 1.4%) for PRL, 0.03 ng/mL (2.5%, 3.9%) for T, and 2 μg/dL (6.5%, 2.7%) for DHEA-S.

DNA was genotyped for 16 KIR genes using PCR-SSOP (sequence-specific oligonucleotide probe) using a commercial kit (LABType KIR SSO Genotyping Test; One Lambda, Canoga Park, CA, USA) and Luminex 100 technology (Austin, TX, USA) as previously described [9].

### Statistical analysis

IBM SPSS Statistics Version 27 (IBM, Tokyo, Japan) was used for statistical analyses. Normality was tested by the Shapiro–Wilk test. If data were not normally distributed, analysis was performed using the Mann–Whitney *U* test or the Wilcoxon matched-pairs signed rank test as appropriate. If data were normally distributed, unpaired *t* tests or paired *t* tests were performed as appropriate. Data also were analyzed using the chi-squared test, Fisher’s exact test, or multiple logistic regression analysis as appropriate. *P* values below 0.05 were considered to indicate significance. Whenever appropriate, results are presented as the mean ± standard deviation (SD).

## Results

### Baseline characteristics of patients

Except for TSH, no significant differences concerning baseline characteristics were evident between the 2 groups completing the study (Table 1). Although TSH was significantly lower in the G-CSF group, these values were within the normal range; neither FT_3_ nor FT_4_ differed between groups, making the TSH difference unlikely to be clinically meaningful. Ranges of AMH values were 0.00 to 1.93 ng/mL in the G-CSF group and 0.00 to 1.77 in the control group. Among the 50 G-CSF patients, 10, 50, 4, and 29 respectively had tubal infertility, ovarian dysfunction, endometriosis, and male infertility, as did 9, 49, 4, and 33 of the 49 control patients, showing no significant differences in prevalence of infertility causes (chi-squared test or Fisher’s exact test).

**Table 1:** Baseline characteristics of patients completing the study ^a^

Characteristic (unit)	G-CSF group (*n* = 50)	Control group (*n* = 49)
Age (years)	36.6 ± 3.8	37.5 ± 3.5
Infertility duration (years)	2.3 ± 2.1	2.4 ± 3.1
Number of previous ART attempts	0.3 ± 0.8	0.2 ± 0.4
Gravidity	0.8 ± 0.8	1.0 ± 1.1
Parity	0.5 ± 0.6	0.4 ± 0.6
Body mass index (kg/m^2^)	20.9 ± 2.3	21.1 ± 2.8
Anti-Müllerian hormone (AMH, ng/mL)	0.98 ± 0.54	0.91 ± 0.49
Follicle-stimulating hormone on day 3 (IU/L)	9.2 ± 6.4	8.6 ± 4.7
Luteinizing hormone on day 3 (IU/L)	4.3 ± 2.7	4.0 ± 1.8
Prolactin (ng/mL)	7.3 ± 3.5	8.8 ± 6.1
Estradiol on day 3 (pg/mL)	32 ± 16	41 ± 28
Free testosterone on day 3 (FT, pg/mL) ^b^	0.60 ± 0.02, *n* = 24	0.60 ± 0.00, *n* = 25
Testosterone on day 3 (T, ng/mL) ^b^	0.16 ± 0.08, *n* = 26	0.17 ± 0.08, *n* = 24
Thyroid-stimulating hormone (TSH, μIU/mL)	1.88 ± 1.07 ^c^	2.46 ± 1.40
Free thyronine (pg/mL)	2.92 ± 0.32	2.84 ± 0.43
Free thyroxine (FT_4_, ng/dL)	1.23 ± 0.14	1.23 ± 0.13
Fasting plasma glucose (mg/dL)	81.9 ± 6.7, *n* = 49	82.9 ± 6.7, *n* = 47
Fasting serum insulin (μU/mL)	4.3 ± 1.9, *n* = 49	4.7 ± 2.0, *n* = 47

^a^ No significant differences were found between groups, except for TSH (unpaired *t* test for T, FT_4_, and AMH; Mann–Whitney *U* test for other characteristics)

^b^ FT and T were measured in the first and second half of this study respectively because production of FT measurement kits was interrupted

^c^*P* < 0.05 *vs.* control group, Mann–Whitney *U* test

### Clinical outcomes

One hundred ART patients were enrolled and randomized to G-CSF or control groups (50 patients each). All G-CSF patients completed the study; 1 control dropped out for unknown reasons, leaving 49 (Fig. 1). No adverse effects of G-CSF were observed. G-CSF and control groups underwent conventional infertility treatments with and without G-CSF priming in the initial and second cycles, resulting in 3 and 0 live deliveries, respectively (Table 2). All 3 patients conceived with spontaneous ovulation after menstruation following the first G-CSF priming.

**Table 2:** Clinical outcomes

Strategy or outcome	G-CSF group	Control group
Patients completing the study	50 patients	49 patients
Conventional infertility treatments in initial and second cycles with or without G-CSF priming		
Live deliveries (% per patient)	3 ^a^ (6.0%)	0 (0%)
IVF/ICSI and fresh ET		
Ovarian stimulation (OS)	47 patients	49 patients
No follicular development induced (% per OS)	0 (0%)	1 (2.0%)
Numbers of follicles (≥ 16 mm) on the hCG day	4.2 ± 2.9	3.0 ± 1.7
Serum E_2_ concentrations (pg/mL) on the hCG day	1820 ± 1200	1350 ± 840
Successful oocyte retrievals (% per OS)	47 (100%)	48 (98%)
No ET for lack of transferrable embryos (% per OS)	2 (4.3%)	4 (8.2%)
Fresh ETs (% per OS)	45 (96%)	44 (90%)
Cryopreservation of redundant blastocysts possible (% per OS)	25 (53%) ^b^	12 (24%)
Clinical pregnancies (% per OS)	14 (30%) ^c^	6 (12%)
Ongoing pregnancies (% per OS)	12 (26%) ^d^	5 (10%)
Live deliveries (% per OS)	9 (19%)	5 (10%)
Cryopreserved ET	21 cycles	14 cycles
Clinical pregnancies (% per cryopreserved ET)	6 (29%)	4 (29%)
Live deliveries (% per cryopreserved ET)	4 (19%)	2 (14%)
Numbers of cumulative live deliveries (% per patient)	16 (32%) ^e^	7 (14%)

^a^ All 3 patients conceived with spontaneous ovulation following initial G-CSF priming

^b^*P* < 0.01 *vs.* control group, chi-squared test; relative risk (RR) = 3.5; 95% confidence interval (CI), 1.5–8.3

^c^*P* < 0.05 *vs.* control group, chi-squared test; RR = 3.0; 95% CI, 1.1–8.8

^d^*P* < 0.05 *vs.* control group, chi-squared test; RR = 3.0; 95% CI, 1.0–9.4

^e^*P* < 0.05 *vs.* control group, chi-squared test; RR = 2.8; 95% CI, 1.04–7.7

Forty-seven G-CSF and forty-nine control patients underwent ovarian stimulation, resulting in successful oocyte retrievals in all G-CSF patients and 48 controls (no follicular growth was induced in one control). No transferable embryos were obtained in 2 G-CSF patients and 4 controls, leaving 45 and 44 fresh ET.

G-CSF and control groups respectively achieved 14 and 6 clinical pregnancies, 12 and 5 ongoing pregnancies, and 9 and 5 live deliveries. Rates of clinical and ongoing pregnancy per stimulated patient were significantly higher in the G-CSF group (30% and 26%) than in controls (12% and 10%, Table 2). Numbers of transferred embryos did not differ significantly between G-CSF and control groups (2.0 ± 0.6 and 1.9 ± 0.4 respectively, *P* = 0.31, Mann–Whitney *U* test). Moreover, significantly more G-CSF patients achieved cryopreservation of redundant blastocysts than controls.

Subsequently, 21 and 14 cycles of cryopreserved ET were carried out in the G-CSF and control groups, resulting in 6 and 4 clinical pregnancies with 4 and 2 live deliveries. Rates of clinical pregnancy per cryopreserved ET were similar between G-CSF and control groups (29% and 29%, *P* = 1.00, Fisher’s exact test), as were numbers of transferred embryos per cryopreserved ET (1.9 ± 0.4 and 1.9 ± 0.3, *P* = 0.52, Mann–Whitney *U* test).

Live delivery rates tended to be higher in the G-CSF group than in controls for any conventional infertility treatment, IVF/ICSI and fresh ET, and cryopreserved ET (6% *vs.* 0%, 19% *vs.* 10%, and 19% *vs.* 14%, respectively), although statistical significance was not attained (*P* = 0.24, Fisher’s exact test; *P* = 0.21, chi-squared test; and *P* = 1.00, Fisher’s exact test). However, the rate of cumulative live delivery per patient was 32% in the G-CSF group, significantly higher than 14% in controls (*P* < 0.05, RR 2.8, 95% CI 1.04–7.7, chi-squared test; Table 2). Miscarriage rates among all clinical pregnancies were similar between the G-CSF and control groups (respectively 30% [7/23] and 30% [3/10]).

Associations of 4 major fertility-related factors (age, day-3 FSH, AMH, and G-CSF priming) with achievement of cumulative live delivery were analyzed by logistic regression analysis. A backward stepwise method based on the likelihood ratio test was used for selection of variables. Only age and G-CSF priming significantly correlated with cumulative live delivery (*P* < 0.05, odds ratio 0.86, 95% CI 0.75–0.99; and *P* < 0.05, odds ratio 2.9, 95% CI 1.0–8.20).

Sixteen G-CSF and seven control patients respectively delivered 20 (10 male, 10 female) and 10 (6 male, 4 female) normal live infants, including 2 and 3 sets of twins and 1 and 0 set of triplets. Considering the 13 and 4 singleton newborns in G-CSF and control groups, no significant difference was evident in body weight (3042 ± 374 *vs.* 2838 ± 648 g; unpaired* t* test), gestational age at delivery (38.5 ± 1.6 *vs.* 37.3 ± 3.1 weeks; unpaired* t* test) or Apgar scores at 1 and 5 min (8.4 ± 0.9 *vs.* 8.0 ± 2.0 and 9.3 ± 0.9 *vs.* 9.3 ± 1.0; Mann–Whitney *U* test).

### Follicular growth, fertilization and embryonic development

On the day of hCG administration, number of follicles larger than 16 mm and serum E_2_ concentrations tended to be higher in 47 G-CSF patients than in 48 controls, although statistical significance was not attained (4.2 ± 2.9 *vs.* 3.0 ± 1.7, *P* = 0.06 and 1820 ± 1200 pg/mL *vs.* 1350 ± 840, *P* = 0.06, respectively; Mann–Whitney *U* test; Table 2). No significant differences (Mann–Whitney *U* test) were evident in endometrial thickness (11.4 ± 2.2 mm *vs.* 11.2 ± 2.6) or total amounts of hMG administered (2800 ± 660 IU *vs.* 2700 ± 810).

**Fig. 2:**
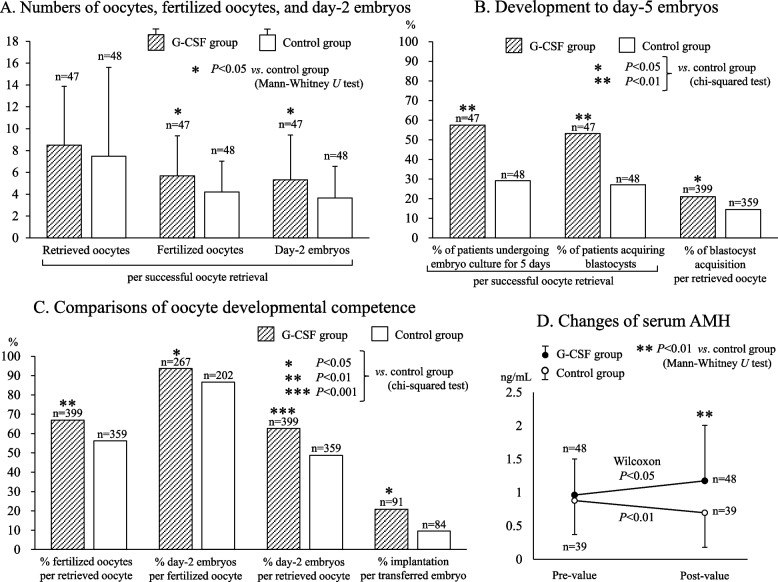
Numbers of retrieved oocytes, fertilized oocytes, and day-2 embryos per successful oocyte retrieval (**A**), development to day-5 embryos (**B**), and oocyte developmental competence (**C**) were compared between G-CSF and control groups. Significantly more fertilized oocytes and day-2 embryos, a higher rate of blastocyst acquisition, and higher embryo quality were obtained in the G-CSF group. Implantation rate per transferred embryo was defined as (number of gestational sacs / number of transferred embryos) × 100%. Serum AMH significantly increased after G-CSF priming; in controls AMH decreased, resulting in higher final concentrations of AMH in the G-CSF group (**D**). G-CSF, granulocyte colony-stimulating factor; AMH, anti-Müllerian hormone

For all patients’retrieved oocytes, developmental outcomes were monitored during the first 2 days of culture. Comparing 47 G-CSF and 48 control patients with successfully retrieved oocytes, numbers of retrieved oocytes did not differ significantly but numbers of fertilized oocytes and day-2 embryos per retrieval were significantly higher in the G-CSF group than in controls (fertilized oocytes, 5.7 ± 3.7 *vs.* 4.2 ± 2.8; day-2 embryos, 5.3 ± 4.1 *vs.* 3.7 ± 2.9; Fig. 2A).

Embryo transfers were carried out at 2, 3, and 5 days after oocyte retrieval in 7, 22, and 18 G-CSF patients and in 9, 27, and 12 control patients, respectively. Distributions of ET days did not differ significantly between groups (chi-squared test). Oocytes from significantly more G-CSF patients proved suitable for embryo culture until 5 days after oocyte retrieval than did oocytes from control patients (57% [27/47] *vs.* 29% [14/48]; Fig. 2B). The rate of blastocyst acquisition per successful oocyte retrieval was significantly higher in the G-CSF group than controls (53% [25/47] *vs.* 27% [13/48]; *P* < 0.01, RR 3.1, 95% CI 1.3–7.2, chi-squared test; Fig. 2B). The frequency of retrieved oocytes developing into blastocysts was also significantly higher for G-CSF patients’ than control patients’ oocytes (21.1% *vs.* 14.5%, Fig. 2B). Consequently, significantly more G-CSF patients achieved cryopreservation of redundant blastocysts than controls (Table 2).

Developmental potentials of oocytes and embryos were significantly greater in the G-CSF group (Fig. 2C). Oocytes were significantly likelier to develop into fertilized oocytes or day-2 embryos in the G-CSF group than in controls. The G-CSF group also showed a significantly higher rate of fertilized oocytes developing into day-2 embryos. Implantation rate per transferred fresh embryo also was significantly higher in the G-CSF group (21% *vs.* 9.5%, *P* < 0.05, RR 2.5, 95% CI 1.03–6.09; chi-squared test; Fig. 2C).

Serum AMH concentrations significantly increased after G-CSF administration but significantly decreased in the same interval for controls (Fig. 2D). Consequently, the latter concentrations of serum AMH were significantly higher in the G-CSF group than in controls (Fig. 2D).

### Genotypes for killer-cell immunoglobulin-like receptor (KIR)

Fisher’s exact test identified no significant differences in frequency of individual KIR genes between patients with and without clinical pregnancies in the G-CSF group (Table 3). Among 2DL5, 2DS1, 2DS5, and 3DS1, none correlated significantly with achievement of clinical pregnancy by G-CSF priming (logistic regression analysis).

**Table 3:** Frequency of individual KIR genes in patients with and without clinical pregnancies in the G-CSF group

KIR genes	With clinical pregnancies (18 patients) No. of patients with each gene (%) ^a^	Without clinical pregnancies (26 patients) No. of patients with each gene (%) ^a^
2DL1	18 (100%)	26 (100%)
2DL2	3 (17%)	6 (23%)
2DL3	18 (100%)	26 (100%)
2DL4	18 (100%)	26 (100%)
2DL5	5 (28%)	5 (19%)
2DP1	18 (100%)	26 (100%)
2DS1	5 (28%)	4 (15%)
2DS2	3 (17%)	6 (23%)
2DS3	2 (11%)	2 (8%)
2DS4	17 (94%)	25 (96%)
2DS5	4 (22%)	3 (12%)
3DL1	17 (94%)	25 (96%)
3DL2	18 (100%)	26 (100%)
3DL3	18 (100%)	26 (100%)
3DP1	18 (100%)	26 (100%)
3DS1	4 (22%)	4 (15%)

^a^ No significant differences in frequency of individual KIR genes were evident between patients with and without clinical pregnancies in the G-CSF group (Fisher’s exact test)

## Discussion

This study suggests a novel, simple, and safe treatment for poor ovarian reserve. In such patients, G-CSF priming in 2 consecutive cycles preceding ART significantly improved fertilization and embryonic development attained by ART, increasing clinical and ongoing pregnancy rates following fresh ET. The cumulative live birth rate was significantly higher in the G-CSF group than in controls. G-CSF priming also significantly increased serum AMH, suggesting enhancement of preantral follicle growth. As G-CSF priming improved oocyte developmental competence without significantly increasing numbers of growing follicles and retrieved oocytes, G-CSF appeared to improve preantral follicle growth in terms of quality rather than quantity. This mechanism clearly differs from those previously suggested for improvement of implantation by G-CSF. We observed no effects of G-CSF priming on miscarriage rates or any association of G-CSF efficacy with KIR genotype.

A variety of clinical effects of G-CSF have been reported. Administration of G-CSF accompanying ET was found to increase implantation rates and clinical pregnancy in ART patients with repeated implantation failure or endometrial thinning [10–14]; such effects have remained uncertain in unselected ART patients [13, 14]. G-CSF also reduced miscarriage rate and increased live birth rate in women with unexplained recurrent miscarriages when its administration was initiated within the implantation window [15], but not when begun following a positive urine pregnancy test [16]. In the absence of 3 activating KIR genes detected particularly frequently in women with unexplained recurrent miscarriage (i.e., lack of 2DS1, 2DS5, and 3DS1) [17], G-CSF has shown high effectiveness in overcoming repeated implantation failure [1]. Intrauterine administration of G-CSF was found to increase endometrial thickness in women with endometrial thinning [2, 18, 19]. Considering such observations, G-CSF administration in the early- and mid-luteal phase may improve endometrial receptivity by immunologic interactions and endometrial growth promotion. G-CSF also can alleviate some forms of ovarian dysfunction; during clomiphene and hCG therapy for infertile patients with luteinized unruptured follicle syndrome, G-CSF administration in the late follicular phase has been found to decrease such follicles [20].

An important difference in our therapeutic use of G-CSF from other reports involves the timing of the result. Previously reported effects of G-CSF occurred promptly, affecting the cycle in which G-CSF was administered. In contrast, we found that G-CSF priming showed novel delayed effects on embryonic development and pregnancy rate in a subsequent cycle. The significant increase in serum AMH and improvement of follicular development in our G-CSF group suggest preantral follicle growth enhancement as an underlying mechanism.

In animal studies, G-CSF attenuated ovarian follicular degeneration and decrements of serum AMH in rats with experimental diabetes [3]. G-CSF also increased numbers of primordial, primary, secondary, and tertiary ovarian follicles in female rats treated with cisplatin [4]. In male mice with acute myeloid leukemia administered chemotherapeutic agents, impaired spermatogenesis and fertility were restored by G-CSF administration [21]. In other experiments, G-CSF administration counteracted apoptosis [22–24], inflammatory states, [3, 21, 24], impaired vascularity [24, 25], growth failure [24, 26], and oxidative stress [3, 24]. Such restoration of a physiologic state might suggest mechanisms applicable to enhancement of preantral follicular growth in our patients with poor ovarian reserve. An autocrine or paracrine role of G-CSF in folliculogenesis might be involved, considering that embryos derived from follicles with higher G-CSF were reported to implant more readily [27].

G-CSF also promotes egress of bone marrow stem cells (BMSC) into peripheral blood [28], potentially aiding tissue regeneration, considering that ovarian transplantation of autologous BMSC collected by apheresis after administration of G-CSF for 5 days was found to improve follicle and oocyte quantity to enable pregnancy in poor ART responders [29]. On the other hand, human plasma derived from apheresis after daily administration of G-CSF for 5 days, which was enriched in BMSC-secreted factors, also improved follicular development and fertility in a mouse model of chemotherapy-induced ovarian damage [30]. Further, through a paracrine action of G-CSF in granulosa cells, human umbilical cord mesenchymal stem cell-derived conditioned medium (hUCMSC-CM) reduced granulosa cell apoptosis and depletion of primordial follicles in cisplatin-treated mice [31]. Thus, an indirect mechanism involving BMSC-secreted factors rather than transdifferentiation of BMSC might be possible.

Clinical safety and tolerance of G-CSF treatment have been established in healthy bone marrow donors treated for 3 to 5 days [26], patients with severe chronic neutropenia treated daily or on alternate days for up to 12 years [32], and patients undergoing ischemic stroke treatment involving G-CSF [24]. In healthy bone marrow donors and patients with repeated implantation failure, unexplained repeated miscarriage, chemotherapy, or severe chronic neutropenia, administration of G-CSF during pregnancy (daily to every 3 days for 1 to 3 trimesters) has shown absence of major maternal or fetal/neonatal adverse effects, including teratogenicity [14–16, 26, 32, 33]. In our study, G-CSF priming had no adverse events in our subjects or their fetuses. All infants born to subjects receiving G-CSF were free of congenital anomalies and had weights similar to those born to controls.

Platelet-rich plasma (PRP) injection into human ovaries has been reported to improve ovarian reserve markers and clinical pregnancy rates [34]. However, ovarian PRP injection with ultrasonographic guidance requires sedation, while subcutaneous G-CSF administration is less invasive, safer, and easier, especially for repeated treatment. We previously administered G-CSF to a 45-year-old perimenopausal ART patient with severely diminished ovarian reserve almost monthly for 3 years (about 31 times), restoring ovulatory cycles without adverse effects. We tentatively chose once-per-cycle administration of G-CSF for our study based upon the serendipitous clinical experience described in the Introduction. However, more frequent administration such as every several days might increase efficacy further; with a single subcutaneous G-CSF administration (100 μg of lenograstim), serum G-CSF concentrations increased from 10.8 ± 4.2 pg/mL on day 0 to 69.6 ± 35.0 on day 1 but fell to 20.9 ± 13.9 on day 6 [20]. In a normal menstrual cycle, serum G-CSF concentrations are lowest in the follicular phase, higher in the luteal phase, and highest in the intervening ovulatory phase [35]. Optimal timing of G-CSF administration during an ovarian cycle remains to be determined.

## Conclusions

In patients with poor ovarian reserve, G-CSF priming in 2 consecutive cycles preceding ART significantly improved fertilization and embryonic development in ART therapy. Consequently, rates of implantation and clinical and ongoing pregnancy by fresh ET were significantly increased. The cumulative live birth rate was significantly higher in the G-CSF group than controls. G-CSF priming also significantly increased serum AMH, consistent with enhancement of preantral follicle growth–a mechanism differing from those previously suggested for implantation improvement by G-CSF. We observed no effects of G-CSF priming on miscarriage rates or any association of its efficacy with KIR genotypes. G-CSF priming showed no adverse events in our subjects or their fetuses. All infants born to subjects receiving G-CSF were free of congenital anomalies and had weights, birth weeks, and Apgar scores similar to those born to controls. This study proposes a novel, simple, and safe treatment for poor ovarian reserve.

## Data Availability

The datasets used and/or analyzed during the current study are available from the corresponding author on reasonable request.

## Abbreviations

AMH: Anti-Müllerian hormone
ART: Assisted reproductive technology
BMSC: Bone marrow stem cells
CI: Confidence interval
DHEA-S: Dehydroepiandrosterone sulfate
ET: Embryo transfer
E_2_: 17β-Estradiol
GnRH: Gonadotropin-releasing hormone
FSH: Follicle-stimulating hormone
FT_3_: Free thyronine
FT_4_: Free thyroxine
G-CSF: Granulocyte colony-stimulating factor
hCG: Human chorionic gonadotropin
hMG: Human menopausal gonadotropin
ICSI: Intracytoplasmic sperm injection
IVF: In vitro Fertilization
KIR: Killer-cell immunoglobulin-like receptor
LH: Luteinizing hormone
OPU: Oocyte pick-up
OS: Ovarian stimulation
PRP: Platelet-rich plasma
PRL: Prolactin
RR: Relative risk
SD: Standard deviation
T: Testosterone
TSH: Thyroid-stimulating hormone
